# Community outbreaks of group A *Streptococcus* revealed by genome sequencing

**DOI:** 10.1038/s41598-017-08914-x

**Published:** 2017-08-17

**Authors:** Claire E. Turner, Luke Bedford, Nicholas M. Brown, Kim Judge, M. Estée Török, Julian Parkhill, Sharon J. Peacock

**Affiliations:** 1 Department of Medicine, Imperial College London, London, United Kingdom; 2 Molecular Biology & Biotechnology and The Florey Institute, University of Sheffield, Sheffield, United Kingdom; 3 Clinical Microbiology and Public Health Laboratory, Cambridge University Hospitals NHS Foundation Trust, Cambridge, United Kingdom; 4 Wellcome Trust Sanger Institute, Cambridge, United Kingdom; 5 Department of Medicine, University of Cambridge, Cambridge, United Kingdom; 6 Cambridge University Hospitals NHS Foundation Trust, Cambridge, United Kingdom; 7 London School of Hygiene and Tropical Medicine, London, United Kingdom

## Abstract

The frequent occurrence of disease outbreaks in humans caused by group A *Streptococcus* (GAS) is an on-going public health threat. Conventional bacterial typing methods lack the discriminatory power to confidently confirm or refute outbreaks in hospital and community settings. Microbial whole genome sequencing (WGS) provides a potential solution to this, but, there has been limited population-based surveillance with accompanying sequence data. We performed retrospective genomic surveillance of 93 clinical GAS isolates from individuals in a defined geographic region. Detailed clinical information was obtained for closely related clusters of isolates. Genomic sequence data was contextualised through comparison with international data. We identified 18 different *emm* genotypes within our bacterial population, and revealed both highly diverse and closely related isolates. This high level of diversity was maintained even in the context of international sequence data. We also identified two *emm*1 clusters, and one *emm*3 cluster, of closely-related isolates that differed only by 1 to 4 single nucleotide polymorphisms. Analysis of clinical information identified no healthcare associated contact between patients, indicating cryptic community transmission. Our findings suggest that genomic surveillance of GAS would increase detection of transmission and highlight opportunities for intervention.

## Introduction

The Lancefield group A *Streptococcus* (GAS; *Streptococcus pyogenes*) is a human pathogen capable of causing a wide spectrum of infections, ranging from self-limiting tonsillitis and pharyngitis to severe, and potentially lethal necrotising fasciitis and toxic shock syndrome. Globally, an estimated 663,000 cases of invasive GAS disease occur per year, resulting in approximately 163,000 deaths^1^. Epidemiological analysis suggests that the overall incidence rate of invasive GAS is increasing^2^, and sporadic increases in global and national prevalence are often associated with specific *emm* genotypes^3–9^. Whole genome sequencing (WGS) data has provided explanations for some of these epidemiological shifts, relating these to changes in gene content. For example, the acquisition of phage carrying novel virulence genes has been associated with a nationwide epidemic of *emm*3 GAS^8^, and homologous recombination of core genomic regions has been linked with a rise in prevalence of a new *emm*89 GAS variant in the UK, Europe and North America^5, 6, 10^.

GAS is also associated with outbreaks of disease. Prior to WGS, probable outbreaks were defined when two or more cases of GAS infection, related by person or place, occurred within a year and isolates shared the same subtypes based on molecular typing^11, 12^. Conventional bacterial typing methods may fail to confirm or refute an outbreak, because of insufficient discrimination between strains of the same lineage. WGS has been used successfully to investigate a small number of putative outbreaks, confirming a single causative strain that was distinct from the circulating population, which were otherwise indistinguishable by standard molecular typing methods^12–14^. Where epidemiological evidence for transmission is unclear or lacking, WGS data could potentially provide valuable supporting evidence to assist outbreak investigations^15^.

The use of WGS in routine practice will require access to contextual genome databases, to enable comparison of outbreak isolates with circulating lineages. This can be used to support decisions on transmission events and outbreaks, may provide more specific information on the abundance of variant strains and disease propensity, and identify cryptic disease clusters occurring in the community. There is currently a paucity of population-based genomic surveillance of GAS and associated sequence data. Here, we describe retrospective genomic surveillance of clinical GAS isolated from individuals in a circumscribed geographic region. This bacterial population contained 18 different *emm* genotypes, and both highly diverse and closely related isolates. The presence of clusters of highly similar isolates with no link to healthcare is indicative of cryptic community transmission.

## Results

We conducted a retrospective observational cohort study at the Cambridge University Hospitals NHS Foundation Trust (CUH) in the UK, and identified 93 patients with at least one GAS isolate stored between 1^st^ January 2006 and 31^st^ December 2012 (Supplementary Table 1). Seventy isolates were from CUH patients, 14 from local district general and community hospitals (GCH), and 9 from general practice (GP). The median age of patients was 37 years (interquartile range; 17 to 67 years), and 47/93 (51%) were male. The majority of isolates were cultured from blood (n = 48) or skin and soft tissue samples (n = 24). The remainder were isolated from throat swabs (n = 7), respiratory secretions (n = 6), bone and joint specimens (n = 1), other sites (n = 1), or unspecified sites (n = 6). Seven patients died as a result of the infective episode within 30 days of the study sample.

**Figure 1:**
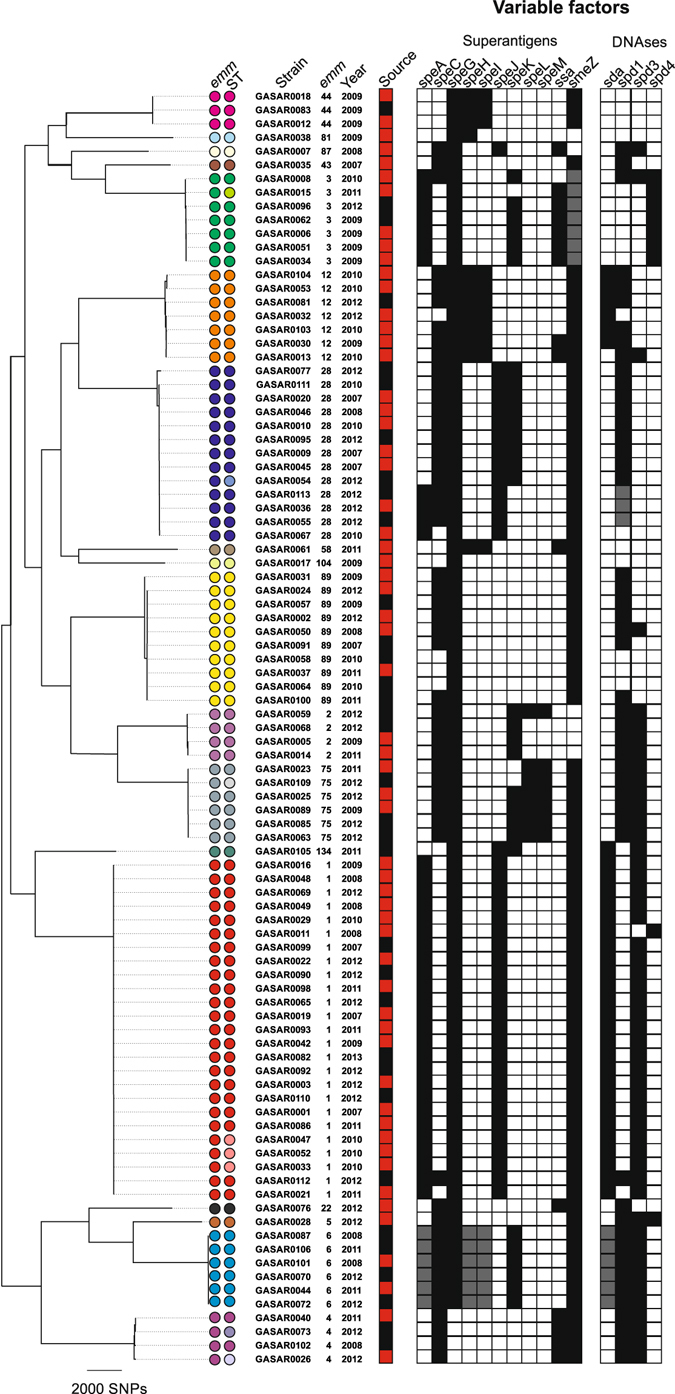
Core gene maximum likelihood phylogenetic analysis of all 93 isolates. All isolates clustered by *emm*-type and there was a broad association of variable factor complement with the *emm*-type. The superantigens *speA*, *speH* and *speI* are highlighted in grey in *emm*6 strains as these are atypical variants that may not be functional. Similarly, all *emm*3 strains were found to carry a non-functional *smeZ* gene, as previously described^16^. The DNase *spd1* in three *emm*28 strains is highlighted grey to indicate that it is a different variant to that found in the other *emm*28 strains. Coloured circles represent *emm*-type/ST. The majority of *emm*-types were associated with a single ST type (colours of *emm*-type identical to colour of ST). Where an additional ST was associated with an *emm*-type this is represent by a different colour ST circle. *emm*1 (n = 25); ST28 (red/red) or ST785 (red/pink), *emm*2 (n = 4); ST55, *emm*3 (n = 7); ST15 (green/green) or ST315 (green/pale green), *emm*4 (n = 4); ST39 (purple/purple) or ST786 (purple/pale purple) or ST38 (purple/paler purple), *emm*6 (n = 6); ST382, *emm*12 (n = 7); ST36, *emm*28 (n = 13); ST52 (blue/blue) or ST787 (blue/pale blue), e*mm*44 (n = 3); ST367, *emm*75 (n = 6); ST150 (grey/grey) or ST788 (grey/paler grey), *emm*89 (n = 10); ST101. Types represented by a single isolate comprised *emm*5; ST99, *emm*22; ST46, *emm*43; ST3 *emm*58; ST176, *emm*81; ST624, *emm*87; ST62, *emm*104; ST789, *emm*134; ST790. Source; Invasive (red) or non-invasive (black) site of isolation.

Eighteen different *emm*-types were derived from sequence data for the 93 isolates (Fig. 1). The most common were *emm*1 (n = 25, 27%), *emm*28 (n = 13, 14%) and *emm*89 (n = 10, 11%), consistent with previous studies from the UK and elsewhere^17–19^. Although patient numbers were small, the mortality associated with *emm*3 was very high, as 3/5 invasive *emm*3 isolates (60%) were associated with attributable death, compared to 2/18 (11%) *emm*1, 1/6 (17%) *emm*12 and 1/8 (13%) *emm*89. Antimicrobial resistance to tetracycline, erythromycin and/or clindamycin was detected in 8 isolates, and was restricted to the less common *emm*-types (Supplementary Table 1). All three *emm*44 isolates and the single *emm*58 isolate carried *tetM* encoding tetracycline resistance, and *ermTR* encoding erythromycin resistance and inducible clindamycin resistance^20^. One of six *emm*75 isolates carried *ermB* and *tetM* and was erythromycin, clindamycin and tetracycline resistant. A further three isolates (*emm*5, *emm*43 and *emm*134) were resistant to tetracycline associated with *tetM*.

The propensity for some strains to cause severe invasive disease has been attributed to loss-of-function mutations within virulence gene regulators, in particular the two component regulatory system CovRS, which negatively regulates a number of different virulence factors^21–23^. We extracted and compared the sequences of known transcriptional regulators, CovR, CovS, RocA (regulator of CovRS), RivR, Rgg1, Rgg2, Rgg3, Rgg4, and FasABCX (Supplementary Tables 2, 3 and 4). A single isolate (an invasive *emm*2) had a mutation in CovR, and 13 isolates, of varying *emm*-type, had mutations in CovS (10 invasive, 3 non-invasive isolates). Although mutations within CovRS have been associated with the virulence and invasive nature of GAS, particularly *emm*1, only two of the 18 invasive *emm*1 isolates had mutations in CovS; an amino acid change of Threonine to Proline at residue 214, and a premature stop codon after 390/500 residues. In addition, one *emm*1 strain (GASAR0033) carried a duplication mutation of 8 bp within *rocA* resulting in a premature stop codon after 32 amino acids, which is predicted to increase virulence factor expression through loss of CovRS repression^24^. None of the isolates from cases who died carried any additional gene regulatory mutations outside of those related to the *emm*-type lineage. Interestingly, 9/13 (69%) *emm*28 isolates carried a mutation in one of five gene regulators; CovS (2 isolates), RivR (1 isolate), Rgg1 (1 isolate), Rgg2 (2 isolates) and Rgg3 (3 isolates). This was a higher prevalence of regulatory gene mutations than other *emm*-types.

A maximum-likelihood tree based on 49,262 SNPs in the 1,295 conserved genes identified by pan-genome analysis identified clustering of isolates by *emm*-type (Fig. 1). Multilocus sequence type (MLST) derived from sequence data resolved 24 STs for the 93 isolates. Each ST contained a single *emm*-type, but five *emm*-types had multiple STs (Fig. 1). In each case the change in ST within an *emm*-type was associated with a single nucleotide base change within one of the seven MLST loci. We also derived *emm*-subtyping information (Supplementary Table 1) but all isolates within each *emm*-type were of the same *emm*-subtype. The exceptions were *emm*1, where the majority were *emm*1.0, but two isolates were *emm*1.52 and two isolates were *emm*1.6, and *emm*6, where the majority were *emm*6.0 but two isolates were *emm*6.4.

Pairwise comparison of SNPs in the core genome of isolates of the same *emm*-type determined a median SNP difference of 37 (range 0–370 SNPs), compared to 11,079 SNP differences (range 8149–13134 SNPs) between isolates of different *emm*-types. The range of zero to 370 SNP differences between isolates within *emm*-types suggest that both identical/very closely related isolates and diverse isolates are circulating within this local population.

The presence of variable accessory genes, which are often associated with mobile prophages, were mapped to the phylogenetic tree (Fig. 1). The pattern of types and subtypes of variable superantigen and DNase genes were predominantly associated with specific *emm*-types, although some were variably present in specific *emm*-type clusters. For example, four of 13 *emm*28 strains were positive for the superantigen *speA* but negative for the superantigen *speK;* both superantigens are associated with different prophages. Three of the same four *emm*28 isolates also had a variant of the DNase *spd1*, commonly found on the same prophage as the superantigen *speC*, that was different from the remaining *emm*28 isolates, and *speC* and *spd1* were both absent in the fourth strain. This suggests that epidemiological studies using detection of these variable accessory genes for isolate discrimination, could be enhanced by including analysis of allelic variants.

### *Emm*1

**Figure 2:**
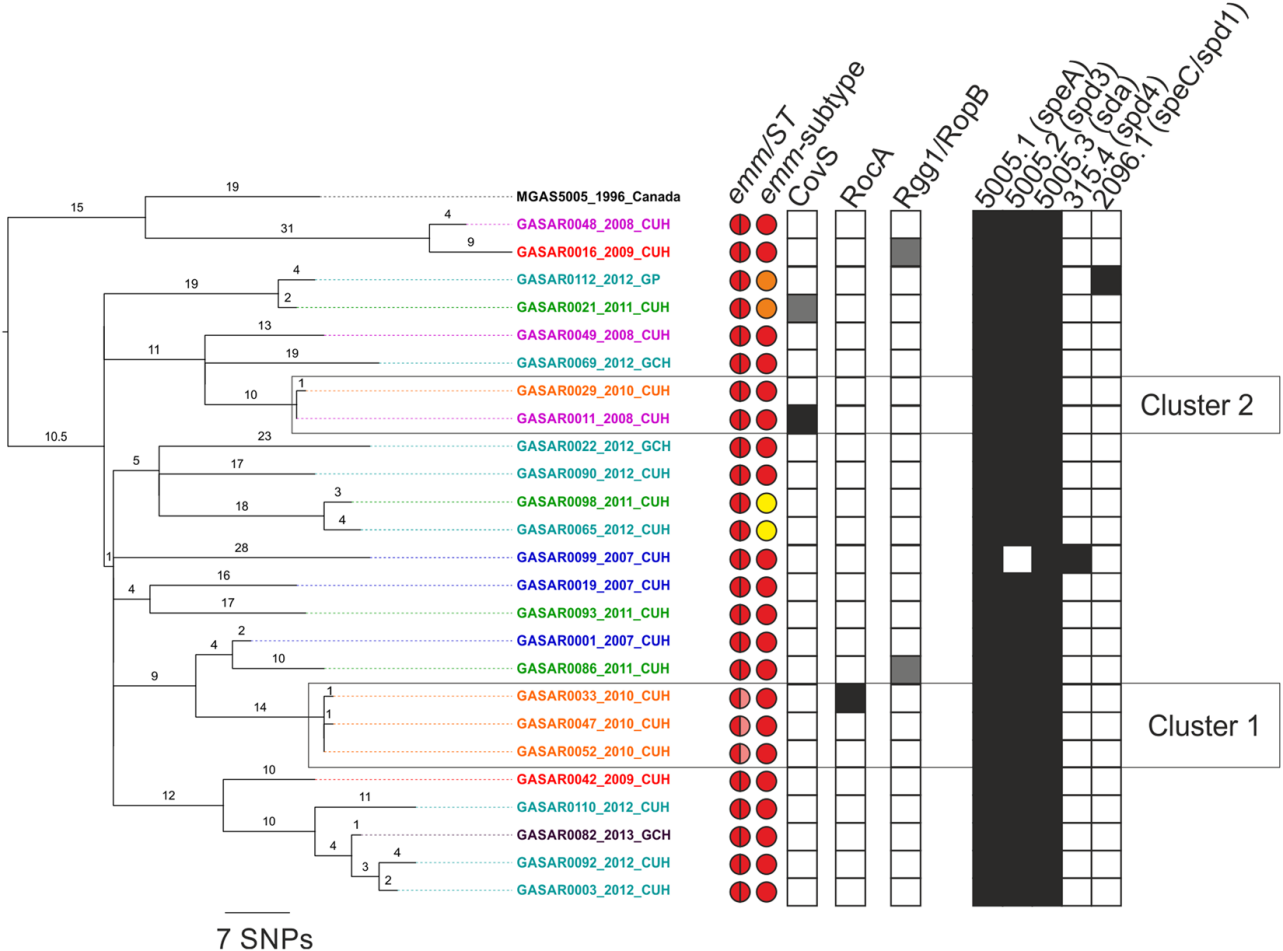
Identification of closely related clusters of *emm*1 isolates. Phylogenetic analysis of 25 *emm*1 isolates mapped to MGAS5005 (NC_007297). Two clusters were identified that were separated by only 1–2 SNPs. Two sequence types were identified: ST28 (red/red) or ST785 (red/pink). All were *emm*-subtype 1.0 except four isolates, of which two were subtype 1.52 (orange) and two were 1.6 (yellow) and the isolates belonging to these subtypes cluster within the phylogeny. Prophages are indicated on the right (presence indicated in black). The majority carried typical M1 prophages 5005.1 with the superantigen *speA*, 5005.2 with the DNase *spd*3 and 5005.3, with the DNase *sda*. GASAR0099 carried a prophage similar to 315.4 from the M3 strain MGAS315 with the DNase *spd4*, and GASAR0112 carried a prophage similar to 2096.1 from the M12 strain MGAS2096 with the superantigen *speC* and the DNase *spd1*. Five isolates had stop codon mutations (in black) or non-synonymous SNP mutations (grey) in three regulatory genes; *covS*, *rocA* and *rgg1/ropB*. Detailed in tip labels are isolate name, year of isolation and site of isolation; Cambridge University Hospital (CUH), Local or tertiary hospitals (GCH) or GP practices (GP). Branches are coloured based on year of isolation and branch numbers indicate SNPs.

We confirmed the presence of two clusters of closely related *emm*1 isolates based on core gene analysis by mapping our *emm*1 population to an *emm*1 reference genome, MGAS5005^25^ (Fig. 2). Cluster 1 contained three isolates, all CUH patients with invasive disease in 2010, and separated by only 1–2 SNPs. GASAR0033 and GASAR0052 were identical by core gene phylogeny (Fig. 1), but mapping to MGAS5005 resolved a unique non-synonymous SNP in GASAR0033 within a non-core gene (serine to leucine at amino acid residue 21 in M5005_Spy0853, short chain dehydrogenase). GASAR0033 also carried an insertion mutation in *rocA*, a regulator of CovRS. A single nucleotide base change in the MLST gene *xpt* of C194T in this cluster of isolates resulted in the unique ST785; other *emm*1 strains were ST28. Two of these patients initially presented with skin and soft tissue infections, and the third patient with bone and joint infection; all went on to develop bacteraemia and multi-organ failure. Patients with skin and soft tissue infections were admitted to the intensive care unit, and one patient died shortly after. GASAR0033 and GASAR0052 were isolated within the same month (22 days apart), although both were community-acquired infections, and GASAR0047 was isolated 7 months later. We were unable to identify any healthcare-related association between any of the three patients.

Cluster 2 contained two invasive isolates from CUH, GASAR0011 (isolated in 2008) and GASAR0029 (isolated in 2010). They were only differentiated by a single non-synonymous mutation in *rplJ* in GASAR0029, but GASAR0011 also carried an insertion mutation in CovS of 25 bp after 1129/1503 bp truncating the protein at 390/500 amino acids, and a deletion mutation in *vfr* (M5005_0693), encoding for a potential virulence factor regulator, truncating the protein at 80/247 amino acids. As these were isolated three years apart, it seems unlikely that they represent direct or recent transmission, but may reflect persistence of this variant in the population.

In addition to Cluster 1 and Cluster 2, three isolate pairs were identified within our collection that were isolated 1–2 years apart and separated by only 6–7 SNPs (Fig. 2); GASAR00112 and GASAR00021, GASAR0098 and GASAR0065, GASAR0092 and GASAR0003. Based on a previously estimated substitution rate for *emm*1 GAS at 1.37 substitutions per core genome per year^3^, these isolate pairs are unlikely to represent recent transmission events, but may represent independent acquisitions of a lineage that is circulating in the community.

**Figure 3:**
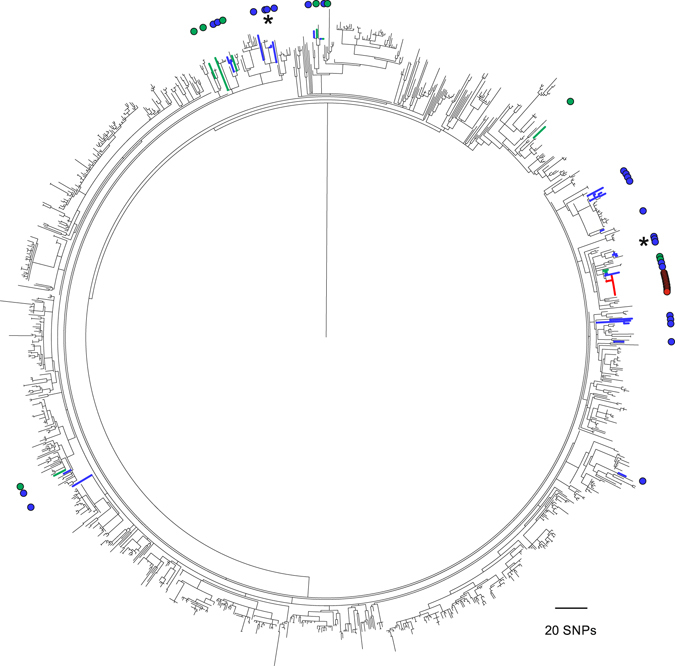
Global context of *emm*1 and outbreak identification. A maximum-likelihood phylogenetic tree was constructed using core genome SNPs (excluding prophages) identified in 1259 sequenced *emm*1 strains isolated 2005–2013 from USA (n = 381), Canada (n = 23), Denmark (n = 269), Finland (n = 204), Norway (n = 54), Iceland (n = 16), Sweden (n = 263)^3^ and the UK (n = 49)^14^ compared to MGAS5005. The UK isolates are identified by coloured branches and highlighted by coloured circles around the outside of the tree. They comprise 25 from the study collection (blue branches and circles), plus 9 from various locations in the UK (green branches and circles) and 15 *emm*1 outbreak isolates (red branches and circles) from a previous study^14^. Although the majority of UK isolates are dispersed across the tree, those associated with the outbreak and the two study local clusters (indicated by *) are clearly distinct from other *emm*1 isolates.

To contextualise our collection, we combined sequence data for the 25 study *emm*1 isolates with data for 1210 *emm*1 isolates from the USA, Canada, Denmark, Finland, Norway, Iceland and Sweden, collected between 2005–2013^3^, and for 24 UK *emm*1 isolates from 2007–2008^14^ (Fig. 3). Cluster 1 and Cluster 2 *emm*1 isolates remained clustered within this extended population. Our other *emm*1 isolates were interspersed with geographically distant isolates across the tree, indicating that the level of diversity in our local population is similar to that of a wider North American/European population. Of the 24 *emm*1 UK isolates sequenced by Turner *et al*.^14^, 15 isolates were part of an outbreak, and were previously determined to be identical to each other but distinct from *emm*1 isolates from other parts of the UK^14^. Incorporation of these 15 outbreak isolates into the extensive collection here supports this finding as they are clearly distinct from other isolates (Fig. 3).

### *Emm*3

**Figure 4:**
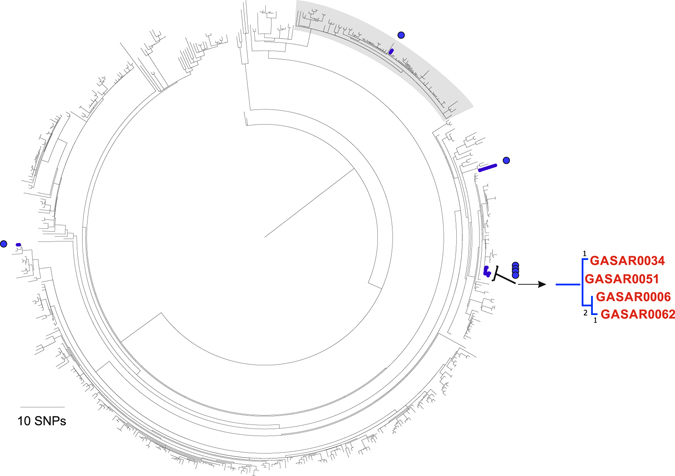
Diverse and closely related isolates within *emm*3. A maximum-likelihood phylogenetic tree was constructed using core genome SNPs (excluding prophages) identified in sequenced *emm*3 strains from our collection (n = 7), *emm*3 strains isolated 1992–2007 from Canada (n = 70)^26^, and *emm*3 strains isolated 2001–2013 from UK and Ireland (n = 442)^8^, compared to the reference *emm*3 genome strain MGAS315. Isolates from our collection are highlighted in blue branches and blue circles around the outside of the tree. One of our isolates resided within the previously identified Lineage C (lineage highlighted grey) associated with an increase in invasive disease in the UK^8^. This strain, like the majority of others within this lineage, carries the superantigen *speC* and the DNase *spd1* but has lost the superantigen *ssa*. An expanded region of the main tree is indicated on the right hand side and highlights four 2009 isolates that are separated from each other by only 1–4 SNPs. Branch labels represent number of SNPs.

Our local collection of seven *emm*3 isolates were associated with high mortality (60%). Like *emm*1, this small population consisted of diverse isolates and a cluster of closely related isolates (Fig. 4). One isolate belonged to a recently identified lineage (Lineage C) associated with an epidemic upsurge in invasive disease in the UK^8^. Four isolates, all obtained in 2009 but isolated one, four and almost 12 months apart from the first isolate, were separated by only 1–4 SNPs (Fig. 4) and two of these isolates (GASAR0006 and GASAR0062) were associated with a fatal outcome. They differed from their nearest relative by two non-synonymous SNPs; threonine to alanine at amino acid residue 112 in *rgpG*, encoding a putative polysaccharide biosynthesis protein, and aspartic acid to asparagine at amino acid residue 588 in *rpoB*, a putative DNA-dependent RNA polymerase subunit β. GASAR0062 also carried a SNP within the −35 transcription box of the *hasA* hyaluronic acid capsule-synthesis gene promoter, which may impact on expression of the hyaluronic acid capsule. GASAR0034 carried a synonymous SNP in *pbp2a* and an 11 bp deletion in CovS, truncating the protein after 221/500 amino acids. Detailed clinical information from all four cases indicated that three patients presented with lower respiratory tract infections and the fourth with pharyngitis (isolate GASAR0034). Three patients developed bacteraemia (isolates GASAR0006, GASAR0034, GASAR0051) and one patient had a skin and soft tissue infection. The relatedness of the strains determined by WGS and the supporting clinical information provides evidence that this was a community outbreak and that it may have extended beyond our cohort.

### *Emm*12

A recent study into the outbreak of scarlet fever in Hong Kong identified four major clades of *emm*12, three of which were associated with scarlet fever isolates^7^. Our local collection of just seven *emm*12 contained isolates from each of the four major clades, suggesting that these clades are a common feature of the *emm*12 population (Supplementary Figure 1). Only two of our isolates carried the superantigen *ssa* which has been attributed to scarlet fever, both were associated with clade I but neither were associated with scarlet fever. Although there has been a recent increase in scarlet fever in the UK, *emm*12 has not yet been identified as a major contributor^27^.

## Discussion

The limited amount of WGS data currently available for GAS means that relatively little is known about the circulating populations in the community. Population-based genomic surveillance of GAS and associated sequence data are crucial in supporting transmission and outbreak investigations. We have identified that even within localised populations there can be both diverse and closely related isolates, indicating both locally circulating clones and the introduction of new lineages.

Analysis of the population core genome identified closely related clusters within the *emm*1 and *emm*3 genotypes that differed only by 0–1 SNPs, indicative of cryptic community transmission. By mapping to a completed reference genome of the same *emm*-type, further differences between isolates were resolved. This suggests that disease clusters can be identified through pan-genome analysis without an *emm*-type specific reference, which is necessary as, although reference genomes have been proven useful, not all *emm*-types are represented. Where disease-clusters are associated with common *emm*-types sharing typical subtypes (MLST, *emm*-subtype, resistance genes), WGS can provide irrefutable evidence of relatedness and identify any unique features that could underpin a specific disease phenotype. In our study, we did not have sufficient numbers to directly link variant isolates or transcriptional regulatory gene mutations with specific clinical markers or disease outcomes. Unusually, however, our *emm*28 isolates did show a higher level of regulatory gene mutations than other *emm*-types, although the relevance of these mutations could not be resolved in our study. With further study and if WGS becomes more routine, is may be possible to tailor treatments towards the specific infection variant.

The majority of invasive disease cases were associated with *emm*1 and mortality was particularly high with *emm*3, making the detection and prevention of disease clusters with these *emm*-types critical. All patients with Cluster 1 *emm*1 isolates went on to develop severe disease, with two requiring ICU admission and one patient died, suggesting this variant may have propensity towards severe invasive disease. Similarly, two of the four closely-related cluster *emm*3 isolates were associated with mortality. These patients with *emm*3 presented with lower respiratory tract infection or pharyngitis, and went on to develop bacteraemia or, in one case, skin and soft tissue infection. Cluster 1 *emm*1 isolates carried a unique MLST and therefore may have been identified through routine typing. The *emm*3 cluster, however, lacked distinguishing conventional typing features (*emm*, MLST, variable accessory factors) suggesting that without whole genome sequencing it would not have been identified. The clinical reports for three of the *emm*3 patients indicated possible contact with other GAS patients outside of this cohort. Although we could not confirm this, it is possible that this cluster extended beyond the four cases that we identified.

There is limited data on GAS transmission and possible infection prevention strategies in the community. Two recent studies identified a 731–2000 fold increased risk of invasive GAS disease among household contacts of a GAS index case compared to the general population, indicating a potential benefit of antibiotic prophylaxis within households^28, 29^. With the rarity of index cases, the number of cases where house-hold or community transmission has been confirmed is very small. Real-time genomic surveillance may provide alerts to outbreak or transmission events and could support the introduction and assessment of community interventions.

We have demonstrated that WGS of isolates within a local population can identify cryptic community transmission and disease clusters. This supports previous findings that WGS of GAS populations can identify disease clusters within the community as well as the spread of unusual GAS genotypes entering the population or the emergence of new GAS variants that may lead to disease upsurges^4–6, 8, 9, 30, 31^. By monitoring local bacterial populations with WGS, informed by circulating national and international genomic data isolates, community transmission could be rapidly identified and interventions put in place. It may also provide early indication of new and potentially harmful variants entering the population that can rapidly become epidemic.

## Methods

### Study Setting, Design and Participants

A retrospective observational cohort study was conducted at the Cambridge University Hospitals NHS Foundation Trust (CUH) in the United Kingdom (UK), a tertiary referral centre in the United Kingdom with 1,170 beds. We identified samples that were culture positive for GAS at the on-site Public Health England Clinical Microbiology and Public Health Laboratory (CMPHL) between 1st January 2006 and 31st December 2012. This diagnostic laboratory receives samples from three hospitals and 75 general practice (GP) surgeries in Cambridgeshire. Isolates were cross-referenced with patient information to identify cases with at least one stored GAS isolate (n = 93). The first stored isolate for each patient was recovered from the laboratory freezer archive for sequencing. Phenotypic antibiotic susceptibility data were obtained from the CMPHL laboratory database. Isolates had previously been tested for susceptibility to penicillin, tetracycline, erythromycin, vancomycin, teicoplanin and clindamycin, using the disc diffusion method (British Society for Antimicrobial Chemotherapy; BSAC methods for Antimicrobial Susceptibility Testing; Version 14 January 2015). Inducible clindamycin resistance was detected using the D-test method.

Clinical data on all 93 cases was collected from paper and electronic patient records, and consisted of date of sample collection, gender, age, infection type/site and 30-day mortality. Seventy isolates were CUH patients, 14 from local district general and community hospitals (GCH), and 9 from general practice (GP). Invasive GAS disease was defined as the isolation of GAS from a normally sterile site or from a wound in a patient with necrotizing fasciitis or streptococcal toxic shock syndrome^32^. Where we suspected clusters of related cases the healthcare records were retrieved and available clinical data was reviewed.

### Whole genome sequencing and analysis

DNA was extracted using the QIAxtractor instrument (QIAgen, Hilden, Germany). DNA library preparation was conducted according to the Illumina protocol and sequencing was performed on an Illumina HiSeq. 2000 with 100-cycle paired-end runs. Sequence data have been submitted to the European Nucleotide Archive (ENA) (www.ebi.ac.uk/ena) under the accession numbers listed in Supplementary Table 5. Genomes were *de novo* assembled using Velvet^33^ with the pipeline and improvements found at https://github.com/sanger-pathogens/vr-codebase and https://github.com/sanger-pathogens/assembly_improvement (assembly statistics in Supplementary Table 5). Genome assemblies were annotated using Prokka and a pan-genome estimated using Roary^34^. A maximum-likelihood tree was created based on 49,262 single nucleotide polymorphisms (SNPs) in the 1,295 conserved genes (present in 100% of isolates with greater than 95% shared identity) using RAxML^35^, a midpoint root and 100 bootstraps. Multilocus sequence types (MLSTs) were identified from the sequence data using the MLST database (pubmlst.org/spyogenes) and an in-house script (https://github.com/sanger-pathogens/mlst_check). *Emm*-types were derived from sequence data and the presence of the 11 known variable chromosomal and prophage-encoded superantigen genes (*speA, speC, speG-M, ssa, smeZ*) and four prophage-encoded DNAse genes (*sda, spd1, spd3, spd4*) determined by BLAST analysis. The sequences of key virulence gene regulators (*covR, covS, rocA, rgg1, rgg2, rgg3, rgg4, fasA, fasB, fasC, fasX, rivR*) were extracted from the assembled genomes and compared to all available reference genomes to identify variant alleles. Within the gene regulators, some genetic variation was identified that was related to *emm*-type, suggesting lineage specific variation and not spontaneous non-functional mutants. We therefore only defined alleles as potential non-functional mutant variants when non-synonymous SNPs occurred in addition to *emm*-type lineage specific variations.

To provide genetic context, sequence data for 1210 *emm*1 isolates from USA/Canada/Europe^3^ and 24 UK *emm*1 isolates^14^ were downloaded from the ENA and combined with the 25 study *emm*1 genomes and mapped to the genome of GAS *emm*1 strain MGAS5005 (ENA accession number NC_007297). Sequence data for 442 *emm*3 GAS isolates from the UK and Ireland^8^ and 70 Canadian *emm*3 isolates^26^ were downloaded from ENA and mapped with the 7 study *emm*3 genomes to *emm*3 strain MGAS315 (ENA accession number NC_004070). 132 *emm*12 isolates from Hong Kong collected between 2005–2011^7^ were downloaded from the ENA and combined with the 7 study *emm*12 isolates and mapped to *emm*12 strain HKU16 (ENA accession number AFRY01000001). All mapping was performed using SMALT (http://www.sanger.ac.uk/resources/software/smalt/), and SNPs in the core genome (excluding prophages) were used to construct a maximum-likelihood tree using RAxML^35^.

### Ethics approval

The study was approved by the National Research Ethics Service (ref: 12/EE/0439) the Cambridge University Hospitals NHS Foundation Trust Research and Development Department (ref: A092428) and we performed the study in accordance with the guidelines and regulations.

### Data availability

Sequence data have been submitted to the European Nucleotide Archive (ENA) (www.ebi.ac.uk/ena) under the accession numbers listed in Supplementary Table 5.

## Electronic supplementary material

Supplementary Tables and Figures

## References

[1] Carapetis JR, Steer AC, Mulholland EK, Weber M (2005). The global burden of group A streptococcal diseases. Lancet Infect Dis..

[2] Sims Sanyahumbi, A., Colquhoun, S., Wyber, R. & Carapetis, J. R. In: Ferretti, J. J., Stevens, D. L. & Fischetti, V. A. editors. *Streptococcus pyogenes*: Basic Biology to Clinical Manifestations. Oklahoma City (OK); 2016.

[3] Nasser W (2014). Evolutionary pathway to increased virulence and epidemic group A *Streptococcus* disease derived from 3,615 genome sequences. Proc Natl Acad Sci USA.

[4] Zhu L (2015). A molecular trigger for intercontinental epidemics of group A *Streptococcus*. J Clin Invest..

[5] Turner CE (2015). Emergence of a new highly successful acapsular group A *Streptococcus* clade of genotype *emm*89 in the United Kingdom. MBio..

[6] Friaes A (2015). Emergence of the same successful clade among distinct populations of *emm*89 *Streptococcus pyogenes* in multiple geographic regions. MBio..

[7] Davies MR (2015). Emergence of scarlet fever *Streptococcus pyogenes emm*12 clones in Hong Kong is associated with toxin acquisition and multidrug resistance. Nat Genet..

[8] Al-Shahib, A. *et al*. Emergence of a novel lineage containing a prophage in *emm*/M3 group A *Streptococcus* associated with upsurge in invasive disease in the UK. *mGen*. **2** (2016). 10.1099/mgen.0.000059 PMC5320645 28348855

[9] Fittipaldi N (2012). Full-genome dissection of an epidemic of severe invasive disease caused by ahypervirulent, recently emerged clone of group A*Streptococcus*. Am J Path..

[10] Zhu L, Olsen RJ, Nasser W, de la Riva Morales I, Musser JM (2015). Trading capsule for increased cytotoxin production: contribution to virulence of a newly emerged clade of *emm*89 *Streptococcus pyogenes*. MBio..

[11] Steer JA (2012). Guidelines for prevention and control of group A streptococcal infection in acute healthcare and maternity settings in the UK. J Infect..

[12] Chalker VJ (2016). Integration of genomic and other epidemiologic data to investigate and control a cross-institutional outbreak of *Streptococcus pyogenes*. Emerg Infect Dis..

[13] Bowen AC (2016). Whole genome sequencing reveals extensive community-level transmission of group A *Streptococcus* in remote communities. Epidemiol Infect..

[14] Turner CE (2013). Molecular analysis of an outbreak of lethal postpartum sepsis caused by *Streptococcus pyogenes*. J Clin Microbiol..

[15] Galloway-Pena J (2016). Application of whole-genome sequencing to an unusual outbreak of invasive group A streptococcal disease. Open Forum Infect Dis..

[16] Turner CE (2012). Superantigenic activity of *emm*3 *Streptococcus pyogenes* is abrogated by a conserved, naturally occurring *smeZ* mutation. PLoS One..

[17] Luca-Harari B (2009). Clinical and microbiological characteristics of severe *Streptococcus pyogenes* disease in Europe. J Clin Microbiol..

[18] Steer AC, Law I, Matatolu L, Beall BW, Carapetis JR (2009). Global *emm*-type distribution of group A streptococci: systematic review and implications for vaccine development. Lancet Infect Dis..

[19] Lamagni TL (2008). Epidemiology of severe *Streptococcus pyogenes* disease in Europe. J Clin Microbiol..

[20] Seppala H, Skurnik M, Soini H, Roberts MC, Huovinen P (1998). A novel erythromycin resistance methylase gene (*ermTR*) in *Streptococcus pyogenes*. Antimicrob Agents Chemother..

[21] Turner CE, Kurupati P, Jones MD, Edwards RJ, Sriskandan S (2009). Emerging role of the interleukin-8 cleaving enzyme SpyCEP in clinical *Streptococcus pyogenes* infection. J Infect Dis..

[22] Sumby P, Whitney AR, Graviss EA, DeLeo FR, Musser JM (2006). Genome-wide analysis of group a streptococci reveals a mutation that modulates global phenotype and disease specificity. PLoS Pathog..

[23] Lin JN, Chang LL, Lai CH, Lin HH, Chen YH (2014). Association between polymorphisms in the *csrRS* two-component regulatory system and invasive group A streptococcal infection. Eur J Clin Microbiol Infect Dis..

[24] Lynskey NN (2013). RocA Truncation Underpins Hyper-Encapsulation, Carriage Longevity and Transmissibility of Serotype M18 Group A Streptococci. PLoS Pathog..

[25] Sumby, P. *et al*. Evolution and emergence of a highly successful clone of serotype M1 group A *Streptococcus* involved multiple horizontal gene transfer events. *J Infect Dis.* **192**, 771–782 (2005). 10.1086/432514 16088826

[26] Beres SB (2010). Molecular complexity of successive bacterial epidemics deconvoluted by comparative pathogenomics. Proc Natl Acad Sci USA.

[27] Turner CE (2016). Scarlet fever upsurge in England and molecular-genetic analysis in North-West London, 2014. Emerg Infect Dis..

[28] Carapetis JR (2014). Effectiveness of clindamycin and intravenous immunoglobulin, and risk of disease in contacts, in invasive group A streptococcal infections. Clin Infect Dis..

[29] Lamagni, T. L., Oliver, I. & Stuart, J. M. Global assessment of invasive group A *Streptococcus* infection risk in household contacts. *Clin Infect Dis.* **60**, 166–7 (2015). 10.1093/cid/ciu752 25258351

[30] Fittipaldi N (2013). Integrated whole-genome sequencing and temporospatial analysis of a continuing group A *Streptococcus* epidemic. Emerg Microbes Infect..

[31] Athey TBT (2016). High incidence of invasive group A *Streptococcus* disease caused by strains of uncommon *emm* types in Thunder Bay, Ontario, Canada. J Clin Micro..

[32] Nelson GE (2016). Epidemiology of invasive group A streptococcal infections in the United States, 2005–2012. Clin Infect Dis..

[33] Zerbino DR, Birney E (2008). Velvet: algorithms for de novo short read assembly using de Bruijn graphs. Genome Res..

[34] Page AJ (2015). Roary: rapid large-scale prokaryote pan genome analysis. Bioinformatics..

[35] Rokas, A. Phylogenetic analysis of protein sequence data using the Randomized Axelerated Maximum Likelihood (RAXML) Program. *Curr Protoc Mol Biol*. Chapter 19 (2011). 10.1002/0471142727.mb1911s96 21987055

