# Supplementary material for: Community outbreaks of group A Streptococcus revealed by genome sequencing
Source: Sci Rep. 2017 Aug 17;7:8554. doi: 10.1038/s41598-017-08914-x (PMC5561225; doi:10.1038/s41598-017-08914-x)
Supplement: Supplementary file 1 — Supplementary Tables and Figures [file 41598_2017_8914_MOESM1_ESM.pdf]

Supplementary Information for:

Community outbreaks of group A *Streptococcus* revealed by genome sequencing

Claire E. Turner, Luke Bedford, Nicholas M. Brown, Kim Judge, M. Estée Török, Julian Parkhill, Sharon J. Peacock

Supplementary Figure 1. Four major clades of *emm*12 represented in our collection.

Supplementary Table 1. List of isolates used in the study

Supplementary Table 2. Transcriptional gene regulator mutations; *covR, covS, rocA, rivR*

Supplementary Table 3. Transcriptional gene regulator mutations; *rgg1, rgg2, rgg3 and rgg4*

Supplementary Table 4. Transcriptional gene regulator mutations; *fasABCX*

Supplementary Table 5. Short read assembly statistics

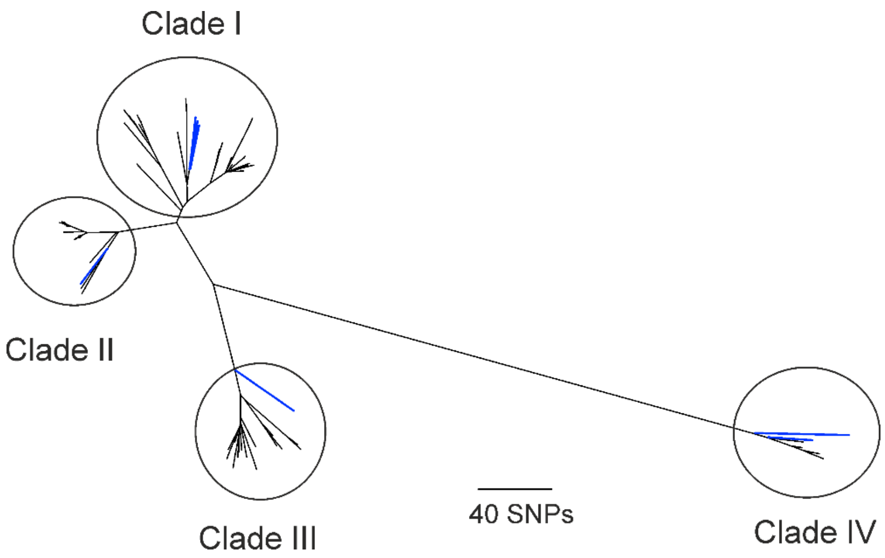

Supplementary Figure 1. Four major clades of *emm*12 represented in our collection. Our seven *emm*12 isolates were mapped to the reference Hong Kong Scarlet fever M12 strain HKU16, along with 132 isolates from Hong Kong collected between 2005-2011[7]. Four main clades identified in the Hong Kong population[7] were represented by at least one isolate from our collection (shown in blue).

Supplementary Table 1. List of isolates used in the study

										Antimicrobial testing[a]						
Sample ID	Referral[b]	Year	Isolate source	Isolate site	*emm* -type	*emm* subtype	ST	*emm*-cluster	30D mortality	Pen	Ery	Tet	Van	Tei	Cli	I-Cli
GASAR0001	CUH	2007	Blood	Invasive	1	1	28	A-C3	No	S	S	S	S	NT	NT	NT
GASAR0002	CUH	2012	Blood	Invasive	89	89	101	E4	No	S	S	S	S	S	S	NT
GASAR0003	CUH	2012	Blood	Invasive	1	1	28	A-C3	No	S	S	S	S	S	S	NT
GASAR0005	CUH	2009	Blood	Invasive	2	2	55	E4	No	S	S	S	S	S	S	NT
GASAR0006	CUH	2009	Blood	Invasive	3	3.1	15	A-C5	**YES**	S	S	S	NT	NT	NT	NT
GASAR0007	CUH	2008	Blood	Invasive	87	87	62	E3	No	S	S	S	S	NT	NT	NT
GASAR0008	CUH	2010	Blood	Invasive	3	3.1	15	A-C5	No	S	S	S	NT	NT	S	NT
GASAR0009	CUH	2007	Blood	Invasive	28	28	52	E4	No	S	S	S	NT	NT	S	NT
GASAR0010	CUH	2010	Blood	Invasive	28	28	52	E4	No	S	S	S	NT	NT	S	NT
GASAR0011	CUH	2008	Blood	Invasive	1	1	28	A-C3	No	S	S	S	S	NT	S	NT
GASAR0012	CUH	2009	Blood	Invasive	44	44	367	E3	No	S	R	R	NT	S	S	Detected
GASAR0013	CUH	2010	Blood	Invasive	12	12	36	A-C4	No	S	S	S	NT	NT	S	NT
GASAR0014	CUH	2011	Blood	Invasive	2	2	55	E4	No	S	S	S	S	NT	S	NT
GASAR0015	CUH	2011	Blood	Invasive	3	3.1	315	A-C5	**YES**	S	S	S	NT	NT	S	NT
GASAR0016	CUH	2009	Blood	Invasive	1	1	28	A-C3	No	S	S	S	S	NT	S	NT
GASAR0017	CUH	2009	Blood	Invasive	104	104	789	E2	No	S	S	S	NT	NT	S	HT
GASAR0018	CUH	2009	Blood	Invasive	44	44	367	E3	No	S	R	R	S	NT	R	NT
GASAR0019	CUH	2007	Blood	Invasive	1	1	28	A-C3	**YES**	S	S	S	S	NT	S	NT
GASAR0020	GCH	2007	Blood	Invasive	28	28	52	E4	No	S	S	S	S	NT	NT	NT
GASAR0021	CUH	2011	Blood	Invasive	1	1.52	28	A-C3	No	S	S	S	S	NT	S	NT
GASAR0022	GCH	2012	Blood	Invasive	1	1	28	A-C3	No	S	S	S	S	S	S	NT
GASAR0023	GCH	2011	Blood	Invasive	75	75	150	E6	No	S	S	S	S	S	S	NT
GASAR0024	GCH	2012	Blood	Invasive	89	89	101	E4	**YES**	S	S	S	S	S	S	NT
GASAR0025	GCH	2012	Blood	Invasive	75	75	150	E6	No	S	S	S	S	S	S	NT
GASAR0026	GCH	2012	Blood	Invasive	4	4	38	E6	No	S	S	S	S	S	S	NT

GASAR0028	CUH	2012	Blood	Invasive	5	5.23	99	Y	No	S	S	R	S	S	S	NT
GASAR0029	CUH	2010	Blood	Invasive	1	1	28	A-C3	No	S	S	S	S	NT	S	NT
GASAR0030	CUH	2009	Blood	Invasive	12	12	36	A-C4	No	S	S	S	S	NT	S	NT
GASAR0031	CUH	2009	Blood	Invasive	89	89	101	E4	No	S	S	S	S	NT	S	NT
GASAR0032	CUH	2012	Blood	Invasive	12	12	36	A-C4	**YES**	S	S	S	S	S	S	NT
GASAR0033	CUH	2010	Blood	Invasive	1	1	785	A-C3	No	S	S	S	S	NT	S	NT
GASAR0034	CUH	2009	Blood	Invasive	3	3.1	15	A-C5	No	S	S	S	S	NT	S	NT
GASAR0035	CUH	2007	Blood	Invasive	43	43.5	3	D4	No	S	S	R	S	NT	S	NT
GASAR0036	GCH	2012	Blood	Invasive	28	28	52	E4	No	S	S	S	S	S	S	NT
GASAR0037	CUH	2011	Blood	Invasive	89	89	101	E4	No	S	S	S	S	S	S	NT
GASAR0038	CUH	2009	Blood	Invasive	81	81	624	E6	No	S	S	S	S	S	S	NT
GASAR0040	CUH	2011	Blood	Invasive	4	4	39	E6	No	S	S	S	NT	NT	S	NT
GASAR0042	CUH	2009	Blood	Invasive	1	1	28	A-C3	No	S	S	S	NT	NT	S	HT
GASAR0044	CUH	2011	Blood	Invasive	6	6.4	382	Y	No	S	S	S	NT	NT	S	NT
GASAR0045	CUH	2007	Blood	Invasive	28	28	52	E4	No	S	S	S	S	NT	S	NT
GASAR0046	GCH	2008	Blood	Invasive	28	28	52	E4	No	S	S	S	S	NT	S	NT
GASAR0047	CUH	2010	Blood	Invasive	1	1	785	A-C3	No	S	S	S	NT	NT	S	NT
GASAR0048	CUH	2008	Blood	Invasive	1	1	28	A-C3	No	S	S	S	S	NT	NT	NT
GASAR0049	CUH	2008	Blood	Invasive	1	1	28	A-C3	No	S	S	S	NT	NT	S	NT
GASAR0050	CUH	2008	Blood	Invasive	89	89	101	E4	No	S	S	S	S	NT	S	NT
GASAR0051	CUH	2009	Blood	Invasive	3	3.1	15	A-C5	No	S	S	S	NT	NT	S	HT
GASAR0052	CUH	2010	Blood	Invasive	1	1	785	A-C3	**YES**	S	S	S	NT	NT	S	NT
GASAR0053	CUH	2010	Blood	Invasive	12	12	36	A-C4	No	S	S	S	NT	NT	S	NT
GASAR0054	GP	2012	SSTI	Non-invasive	28	28	787	E4	No	S	S	S	S	S	S	NT
GASAR0055	GP	2012	SSTI	Non-invasive	28	28	52	E4	No	S	S	S	S	S	S	NT
GASAR0057	CUH	2009	Respiratory	Non-invasive	89	89	101	E4	No	S	S	S	NT	NT	S	NT
GASAR0058	CUH	2010	SSTI	Non-invasive	89	89	101	E4	No	S	S	S	NT	NT	S	NT
GASAR0059	GP	2012	SSTI	Non-invasive	2	2	55	E4	No	S	S	S	S	S	S	NT
GASAR0061	CUH	2011	Unspecified	Invasive	58	58	176	E3	No	S	R	R	S	S	S	Detected

GASAR0062	CUH	2009	SSTI	Non-invasive	3	3.1	15	A-C5	**YES**	S	S	S	NT	NT	S	NT
GASAR0063	GP	2012	SSTI	Non-invasive	75	75	150	E6	No	S	S	S	S	S	S	NT
GASAR0064	CUH	2010	SSTI	Non-invasive	89	89	101	E4	No	S	S	S	NT	NT	S	NT
GASAR0065	CUH	2012	SSTI	Non-invasive	1	1.6	28	A-C3	No	S	S	S	S	S	S	NT
GASAR0067	CUH	2010	SSTI	Invasive	28	28	52	E4	No	S	S	S	NT	NT	S	NT
GASAR0068	CUH	2012	Throat	Non-invasive	2	2	55	E4	No	S	S	S	S	S	S	NT
GASAR0069	GCH	2012	Respiratory	Invasive	1	1	28	A-C3	No	S	S	S	S	S	S	NT
GASAR0070	GCH	2012	Throat	Non-invasive	6	6	382	Y	No	S	S	S	S	S	S	NT
GASAR0072	GCH	2012	Throat	Non-invasive	6	6.4	382	Y	No	S	S	S	S	S	S	NT
GASAR0073	GCH	2012	Throat	Non-invasive	4	4	786	E6	No	S	S	S	S	S	S	NT
GASAR0076	GCH	2012	SSTI	Non-invasive	22	22	46	E4	No	S	S	S	S	S	S	NT
GASAR0077	GCH	2012	SSTI	Non-invasive	28	28	52	E4	No	S	S	S	S	S	S	NT
GASAR0081	GP	2012	SSTI	Non-invasive	12	12	36	A-C4	No	S	S	S	S	S	S	NT
GASAR0082	GCH	2013	SSTI	Non-invasive	1	1	28	A-C3	No	S	S	S	S	S	S	NT
GASAR0083	GP	2009	SSTI	Non-invasive	44	44	367	E3	No	S	R	R	NT	NT	S	Detected
GASAR0085	CUH	2012	SSTI	Non-invasive	75	75	150	E6	No	S	R	R	S	S	R	NT
GASAR0086	CUH	2011	Bone+joint	Invasive	1	1	28	A-C3	No	S	S	S	S	S	S	NT
GASAR0087	CUH	2008	Respiratory	Non-invasive	6	6	382	Y	No	S	S	S	NT	NT	S	NT
GASAR0089	CUH	2009	SSTI	Invasive	75	75	150	E6	No	S	S	S	NT	NT	S	NT
GASAR0090	CUH	2012	Unspecified	Non-invasive	1	1	28	A-C3	No	S	S	S	S	S	S	NT
GASAR0091	CUH	2007	SSTI	Non-invasive	89	89	101	E4	No	S	S	S	NT	NT	S	NT
GASAR0092	CUH	2012	Throat	Non-invasive	1	1	28	A-C3	No	S	S	S	S	S	S	NT
GASAR0093	CUH	2011	SSTI	Invasive	1	1	28	A-C3	No	S	S	S	NT	NT	S	NT
GASAR0095	GP	2012	SSTI	Non-invasive	28	28	52	E4	No	S	S	S	S	S	S	NT
GASAR0096	CUH	2012	SSTI	Non-invasive	3	3.1	15	A-C5	No	S	S	S	S	S	S	NT
GASAR0098	CUH	2011	SSTI	Invasive	1	1.6	28	A-C3	No	S	S	S	NT	NT	S	NT
GASAR0099	CUH	2007	Unspecified	Non-invasive	1	1	28	A-C3	No	S	S	S	NT	NT	S	NT
GASAR0100	CUH	2011	Respiratory	Non-invasive	89	89	101	E4	No	S	S	S	S	S	S	NT
GASAR0101	CUH	2008	Other	Invasive	6	6	382	Y	No	S	S	S	NT	S	S	NT

GASAR0102	CUH	2008	SSTI	Non-invasive	4	4	39	E6	No	S	S	S	NT	NT	S	NT
GASAR0103	CUH	2010	Respiratory	Invasive	12	12	36	A-C4	No	S	S	S	NT	NT	S	NT
GASAR0104	CUH	2010	Respiratory	Invasive	12	12	36	A-C4	No	S	S	S	NT	NT	S	NT
GASAR0105	CUH	2011	SSTI	Non-invasive	134	134	790	E5	No	S	S	R	NT	NT	S	NT
GASAR0106	CUH	2011	Unspecified	Non-invasive	6	6	382	Y	No	S	S	S	NT	NT	S	NT
GASAR0109	CUH	2012	Throat	Non-invasive	75	75	788	E6	No	S	S	S	S	S	S	NT
GASAR0110	CUH	2012	Unspecified	Non-invasive	1	1	28	A-C3	No	S	S	S	S	S	S	NT
GASAR0111	GCH	2010	Unspecified	Non-invasive	28	28	52	E4	No	S	S	S	NT	NT	S	NT
GASAR0112	GP	2012	SSTI	Non-invasive	1	1.52	28	A-C3	No	S	S	S	S	S	S	NT
GASAR0113	GP	2012	Throat	Non-invasive	28	28	52	E4	No	S	S	S	S	S	S	NT

[a] Pen; Penicillin, Ery; Erythromycin, Tet; Tetracycline, Van; Vancomycin, Tec; Teicoplanin, Cli; Clindamycin, I-Cli; Inducible clindamycin. NT; not tested.

[b] Referral from Cambridge University Hospital (CUH), Local tertiary and general care hospitals (GCH), General Practice (GP).

Supplementary Table 2. Transcriptional gene regulator mutations; *covR*, *covS*, *rocA*, *rivR*

		covR			***covS***			***rocA***			***rivR***		
**Strain**	***emm* -type**	***emm*-specific**	**Other**	**Mutant**	***emm*-specific**	**Other**	**Mutant**	***emm*-specific**	**Other**	**Mutant**	***emm*-specific**	**Other**	**Mutant**
GASAR0001	1	-	-		I332V[a]	-		Q259R	-		E420K	-	
GASAR0003	1	-	-		I332V	-		Q259R	-		E420K	-	
GASAR0011	1	-	-		I332V	**390-STOP**	**Yes**	Q259R	-		E420K	-	
GASAR0016	1	-	-		I332V	-		Q259R	-		E420K	-	
GASAR0019	1	-	-		I332V	-		Q259R	-		E420K	-	
GASAR0021	1	-	-		I332V	**T214P**	**Yes**	Q259R	-		E420K	-	
GASAR0022	1	-	-		I332V	-		Q259R	-		E420K	-	
GASAR0029	1	-	-		I332V	-		Q259R	-		E420K	-	
GASAR0033	1	-	-		I332V	-		Q259R	**32-STOP**	**Yes**	E420K	-	
GASAR0042	1	-	-		I332V	-		Q259R	-		E420K	-	
GASAR0047	1	-	-		I332V	-		Q259R	-		E420K	-	
GASAR0048	1	-	-		I332V	-		Q259R	-		E420K	-	
GASAR0049	1	-	-		I332V	-		Q259R	-		E420K	-	
GASAR0052	1	-	-		I332V	-		Q259R	-		E420K	-	
GASAR0065	1	-	-		I332V	-		Q259R	-		E420K	-	
GASAR0069	1	-	-		I332V	-		Q259R	-		E420K	-	
GASAR0082	1	-	-		I332V	-		Q259R	-		E420K	-	
GASAR0086	1	-	-		I332V	-		Q259R	-		E420K	-	
GASAR0090	1	-	-		I332V	-		Q259R	-		E420K	-	
GASAR0092	1	-	-		I332V	-		Q259R	-		E420K	-	
GASAR0093	1	-	-		I332V	-		Q259R	-		E420K	-	
GASAR0098	1	-	-		I332V	-		Q259R	-		E420K	-	
GASAR0099	1	-	-		I332V	-		Q259R	-		E420K	-	
GASAR0110	1	-	-		I332V	-		Q259R	-		E420K	-	
GASAR0112	1	-	-		I332V	-		Q259R	-		E420K	-	
GASAR0005	2	-	**R66C**	**Yes**	-	-		-	-		K101R; M345K	-	

GASAR0014	2	-	-	-	**E317D**	**Yes**	-	-		K101R; M345K	-	
GASAR0059	2	-	-	-	-		-	-		K101R; M345K	-	
GASAR0068	2	-	-	-	-		-	-		K101R; M345K	-	
GASAR0006	3	-	-	-	-		I203N, L247F, D396N, I404T, **417-STOP**	-		**89-STOP**	-	
GASAR0008	3	-	-	-	**46-STOP**	**Yes**	I203N, L247F, D396N, I404T, **417-STOP**	-		**89-STOP**	-	
GASAR0015	3	-	-	-	-		I203N, L247F, D396N, I404T, **417-STOP**	-		**89-STOP**	-	
GASAR0034	3	-	-	-	**221-STOP**	**Yes**	I203N, L247F, D396N, I404T, **417-STOP**	-		**89-STOP**	-	
GASAR0051	3	-	-	-	-		I203N, L247F, D396N, I404T, **417-STOP**	-		**89-STOP**	-	
GASAR0062	3	-	-	-	-		I203N, L247F, D396N, I404T, **417-STOP**	-		**89-STOP**	-	
GASAR0096	3	-	-	-	**407-STOP**	**Yes**	I203N, L247F, D396N, I404T, **417-STOP**	-		**89-STOP**	-	
GASAR0026	4	-	-	K498N	-		V182I, D396N, I404T	-		**192-STOP**	-	
GASAR0040	4	-	-	K498N	-		V182I, D396N, I404T	-		**192-STOP**	-	
GASAR0073	4	-	-	K498N	-		V182I, D396N, I404T	-		**192-STOP**	-	
GASAR0102	4	-	-	K498N	-		V182I, D396N, I404T	-		**192-STOP**	-	
GASAR0028	5	-	-	K498N	-	?[b]	V47A, D396N, I404T, P405S	-	?	Q281H; G465S	-	?
GASAR0044	6	-	-	M228I, G357D, K498N	-		V346L, D396N, I404T	-		-	-	
GASAR0070	6	-	-	M228I, G357D, K498N	-		V346L, D396N, I404T	-		-	-	
GASAR0072	6	-	-	M228I, G357D, K498N	-		V346L, D396N, I404T	-		-	-	
GASAR0087	6	-	-	M228I, G357D, K498N	-		V346L, D396N, I404T	-		-	-	
GASAR0101	6	-	-	M228I, G357D, K498N	-		V346L, D396N, I404T	-		-	-	
GASAR0106	6	-	-	M228I, G357D, K498N	-		V346L, D396N, I404T	-		-	-	

GASAR0013	12	-	-	-	-		-	-		V500I	-	
GASAR0030	12	-	-	-	-		-	-		V500I	-	
GASAR0032	12	-	-	-	-		-	-		V500I	-	
GASAR0053	12	-	-	-	-		-	-		V500I	-	
GASAR0081	12	-	-	-	-		-	-		V500I	-	
GASAR0103	12	-	-	-	-		-	-		V500I	-	
GASAR0104	12	-	-	-	-		-	-		V500I	-	
GASAR0076	22	-	-	E265D, E428K		?	V333A	-	?	Q487H	-	
GASAR0009	28	-	-	E226G	-		D396N, I404T	-		Q278R; R279Q; A407G	-	
GASAR0010	28	-	-	E226G	-		D396N, I404T	-		Q278R; R279Q; A407G	**M91V**	**Yes**
GASAR0020	28	-	-	E226G	-		D396N, I404T	-		Q278R; R279Q; A407G	-	
GASAR0036	28	-	-	E226G	-		D396N, I404T	-		Q278R; R279Q; A407G	-	
GASAR0045	28	-	-	E226G	-		D396N, I404T	-		Q278R; R279Q; A407G	-	
GASAR0046	28	-	-	E226G	**P220S**	**Yes**	D396N, I404T	-		Q278R; R279Q; A407G	-	
GASAR0054	28	-	-	E226G	-		D396N, I404T	-		Q278R; R279Q; A407G	-	
GASAR0055	28	-	-	E226G	-		D396N, I404T	-		Q278R; R279Q; A407G	-	
GASAR0067	28	-	-	E226G	-		D396N, I404T	-		Q278R; R279Q; A407G	-	
GASAR0077	28	-	-	E226G	-		D396N, I404T	-		Q278R; R279Q; A407G	-	
GASAR0095	28	-	-	E226G	-		D396N, I404T	-		Q278R; R279Q; A407G	-	
GASAR0111	28	-	-	E226G	**R104C**	**Yes**	D396N, I404T	-		Q278R; R279Q; A407G	-	
GASAR0113	28	-	-	E226G	-		D396N, I404T	-		Q278R; R279Q; A407G	-	
GASAR0035	43	-	-	-	-		V182I, D396N, I404T	-	?	K101R; G455S	-	?
GASAR0012	44	-	-	P493A	-		F349L	-		-	-	
GASAR0018	44	-	-	P493A	-		F349L	-		-	-	
GASAR0083	44	-	-	P493A	-		F349L	-		-	-	
GASAR0061	58	-	-	-	-		I203N, D396N, I404T	-	?	Q281H	-	?

GASAR0023	75	-	-	S337L	-		-	-		K101R; M345K	-	
GASAR0025	75	-	-	S337L	**46-STOP**	**Yes**	-	-		K101R; M345K	-	
GASAR0063	75	-	-	S337L	-		-	-		K101R; M345K	-	
GASAR0085	75	-	-	S337L	-		-	-		K101R; M345K	-	
GASAR0089	75	-	-	S337L	**496-STOP**	**Yes**	-	-		K101R; M345K	-	
GASAR0109	75	-	-	S337L	-		-	-		K101R; M345K	-	
GASAR0038	81	-	-	-	-		-	-		D193G	-	?
GASAR0007	87	-	-	-	-		D310A, L302S	-	?	-	-	
GASAR0002	89	-	-	-	-		-	-		-	-	
GASAR0024	89	-	-	-	-		-	-		-	-	
GASAR0031	89	-	-	-	**46-STOP**	**Yes**	-	-		-	-	
GASAR0037	89	-	-	-	-		-	-		-	-	
GASAR0050	89	-	-	-	-		-	-		-	-	
GASAR0057	89	-	-	-	-		-	-		-	-	
GASAR0058	89	-	-	-	-		-	-		-	-	
GASAR0064	89	-	-	-	-		-	-		-	-	
GASAR0091	89	-	-	-	**P136S**	**Yes**	-	-		-	-	
GASAR0100	89	-	-	-	-		-	-		-	-	
GASAR0017	104	-	-	-	**46-STOP**	**Yes**	D310A, L302S	-	?	K101R	-	?
GASAR0105	134	-	-	-	-		I404T	-	?	M345K	-	?

[a] Amino acid change at the indicated residue compared to the amino acid sequences for each gene obtained from all the isolates

[b] ?; Too few strains representing the *emm*-type to determine between *emm*-type associated variations and additional mutations.

Supplementary Table 3. Transcriptional gene regulator mutations; *rgg1, rgg2, rgg3 and rgg4*

		rgg1 (ropB)			*rgg2*			*rgg3*			*rgg4*			
**Strain**	***emm* -type**	***emm*-specific**	**Other**	**Mutant**	***emm*-specific**	**Other**	**Mutant**	***emm*-specific**	**Other**	**Mutant**	**Type[a]**	***emm*-specific**	**Other**	**Mutant**
GASAR0001	1	-	-		-	-		-	-		1	-	-	
GASAR0003	1	-	-		-	-		-	-		1	-	-	
GASAR0011	1	-	-		-	-		-	-		1	-	-	
GASAR0016	1	-	**D40G**	**Yes**	-	-		-	-		1	-	-	
GASAR0019	1	-	-		-	-		-	-		1	-	-	
GASAR0021	1	-	-		-	-		-	-		1	-	-	
GASAR0022	1	-	-		-	-		-	-		1	-	-	
GASAR0029	1	-	-		-	-		-	-		1	-	-	
GASAR0033	1	-	-		-	-		-	-		1	-	-	
GASAR0042	1	-	-		-	-		-	-		1	-	-	
GASAR0047	1	-	-		-	-		-	-		1	-	-	
GASAR0048	1	-	-		-	-		-	-		1	-	-	
GASAR0049	1	-	-		-	-		-	-		1	-	-	
GASAR0052	1	-	-		-	-		-	-		1	-	-	
GASAR0065	1	-	-		-	-		-	-		1	-	-	
GASAR0069	1	-	-		-	-		-	-		1	-	-	
GASAR0082	1	-	-		-	-		-	-		1	-	-	
GASAR0086	1	-	**E8G**	**Yes**	-	-		-	-		1	-	-	
GASAR0090	1	-	-		-	-		-	-		1	-	-	
GASAR0092	1	-	-		-	-		-	-		1	-	-	
GASAR0093	1	-	-		-	-		-	-		1	-	-	
GASAR0098	1	-	-		-	-		-	-		1	-	-	
GASAR0099	1	-	-		-	-		-	-		1	-	-	
GASAR0110	1	-	-		-	-		-	-		1	-	-	
GASAR0112	1	-	-		-	-		-	-		1	-	-	
GASAR0005	2	-	-		E239K; M241I	-		A7V	-		2	V183A	-	

GASAR0014	2	-	-		E239K; M241I	-		A7V	-		2	V183A	-
GASAR0059	2	-	-		E239K; M241I	-		A7V	**D274N**	**Yes**	2	V183A	-
GASAR0068	2	-	-		E239K; M241I	-		A7V	-		2	V183A	-
GASAR0006	3	-	-		-	-		E144K	-		2	R227H	-
GASAR0008	3	-	-		-	-		E144K	-		2	R227H	-
GASAR0015	3	-	-		-	-		E144K	-		2	R227H	-
GASAR0034	3	-	-		-	-		E144K	-		2	R227H	-
GASAR0051	3	-	-		-	-		E144K	-		2	R227H	-
GASAR0062	3	-	-		-	-		E144K	-		2	R227H	-
GASAR0096	3	-	-		-	-		E144K	-		2	R227H	-
GASAR0026	4	-	-		E239K; M241I	-		-	-		1	I266V	-
GASAR0040	4	-	**T104I**	**Yes**	E239K; M241I	-		-	-		1	I266V	-
GASAR0073	4	-	-		E239K; M241I	-		-	-		1	I266V	-
GASAR0102	4	-	-		E239K; M241I	-		-	-		1	I266V	-
GASAR0028	5	V169I	-	?	E239K; M241I; D280G	-	?	A7V	-	?	2	-	-
GASAR0044	6	-	-		E239K; M241I	-		A7V	-		1	-	-
GASAR0070	6	-	-		E239K; M241I	-		A7V	-		1	-	-
GASAR0072	6	-	-		E239K; M241I	-		A7V	-		1	-	-
GASAR0087	6	-	**G90C**	**Yes**	E239K; M241I	-		A7V	-		1	-	-
GASAR0101	6	-	-		E239K; M241I	-		A7V	-		1	-	-
GASAR0106	6	-	-		E239K; M241I	-		A7V	-		1	-	-
GASAR0013	12	E89D	-		-	-		-	-		1	-	-
GASAR0030	12	E89D	**E72K**	**Yes**	-	-		-	-		1	-	-
GASAR0032	12	E89D	-		-	-		-	-		1	-	-
GASAR0053	12	E89D	-		-	-		-	-		1	-	-
GASAR0081	12	E89D	-		-	-		-	-		1	-	-
GASAR0103	12	E89D	-		-	-		-	-		1	-	-
GASAR0104	12	E89D	-		-	-		-	-		1	-	-
GASAR0076	22	-	-		-	-		-	-		1	-	-

GASAR0009	28	-	**V21A**	**Yes**	-	-		T219S	-		1	-	-	
GASAR0010	28	-	-		-	-		T219S	-		1	-	-	
GASAR0020	28	-	-		-	-		T219S	-		1	-	-	
GASAR0036	28	-	-		-	-		T219S	**F198L**	**Yes**	1	-	-	
GASAR0045	28	-	-		-	-		T219S	-		1	-	-	
GASAR0046	28	-	-		-	-		T219S	-		1	-	-	
GASAR0054	28	-	-		-	-		T219S	-		1	-	-	
GASAR0055	28	-	-		-	**A100D**	**Yes**	T219S	-		1	-	-	
GASAR0067	28	-	-		-	**A100D**	**Yes**	T219S	-		1	-	-	
GASAR0077	28	-	-		-	-		T219S	**H27Y**	**Yes**	1	-	-	
GASAR0095	28	-	-		-	-		T219S	-		1	-	-	
GASAR0111	28	-	-		-	-		T219S	-		1	-	-	
GASAR0113	28	-	-		-	-		T219S	**F198L**	**Yes**	1	-	-	
GASAR0035	43	-	-		-	-		-	-		1	-	-	
GASAR0012	44	-	**F62S**	**Yes**	-	-		-	-		2	N267S	-	
GASAR0018	44	-	-		-	-		-	-		2	N267S	-	
GASAR0083	44	-	-		-	-		-	-		2	N267S	-	
GASAR0061	58	-	-		E239K; M241I	-	?	T219S	-	?	1	-	-	
GASAR0023	75	-	-		E239K; M241I	-		-	-		2	V183A	-	
GASAR0025	75	-	-		E239K; M241I	-		-	-		2	V183A	-	
GASAR0063	75	-	-		E239K; M241I	-		-	-		2	V183A	-	
GASAR0085	75	-	-		E239K; M241I	-		-	-		2	V183A	**D91N**	**Yes**
GASAR0089	75	-	-		E239K; M241I	-		-	-		2	V183A	-	
GASAR0109	75	-	-		E239K; M241I	-		-	-		2	V183A	-	
GASAR0038	81	-	-		-	-		T219S	-	?	2	S201P	-	
GASAR0007	87	-	-		-	-		-	-		1	-	-	
GASAR0002	89	-	-		-	-		E68K; T219S	-		1	-	-	
GASAR0024	89	-	-		-	-		E68K; T219S	-		1	-	-	
GASAR0031	89	-	**217-STOP**	**Yes**	-	-		E68K; T219S	-		1	-	-	

GASAR0037	89	-	-		-	-		E68K; T219S	-		1	-	-
GASAR0050	89	-	-		-	-		E68K; T219S	-		1	-	-
GASAR0057	89	-	**C222F**	**Yes**	-	-		E68K; T219S	-		1	-	-
GASAR0058	89	-	-		-	-		E68K; T219S	-		1	-	-
GASAR0064	89	-	-		-	-		E68K; T219S	-		1	-	-
GASAR0091	89	-	-		-	-		E68K; T219S	-		1	-	-
GASAR0100	89	-	-		-	-		E68K; T219S	-		1	-	-
GASAR0017	104	-	-		E239K; M241I	-	?	I199V	-	?	1	-	-
GASAR0105	134	-	-		-	-		T219S	-	?	1	-	-

[a] There are two types of *rgg*4 based on the DNA sequence

Supplementary Table 4. Transcriptional gene regulator mutations; *fasABCX*

		fasA			*fasB*			*fasC*			*fasX* (DNA sequence)		
**Strain**	***emm* -type**	***emm*-specific**	**Other**	**Mutant**	***emm*-specific**	**Other**	**Mutant**	***emm*-specific**	**Other**	**Mutant**	***emm*-specific**	**Other**	**Mutant**
GASAR0001	1	-	-		T237I; D375G	-		-	-		-	-	
GASAR0003	1	-	-		T237I; D375G	-		-	-		-	-	
GASAR0011	1	-	-		T237I; D375G	-		-	-		-	-	
GASAR0016	1	-	-		T237I; D375G	-		-	-		-	-	
GASAR0019	1	-	-		T237I; D375G	-		-	-		-	-	
GASAR0021	1	-	-		T237I; D375G	-		-	-		-	-	
GASAR0022	1	-	-		T237I; D375G	-		-	-		-	-	
GASAR0029	1	-	-		T237I; D375G	-		-	-		-	-	
GASAR0033	1	-	-		T237I; D375G	-		-	-		-	-	
GASAR0042	1	-	-		T237I; D375G	-		-	-		-	-	
GASAR0047	1	-	-		T237I; D375G	-		-	-		-	-	
GASAR0048	1	-	-		T237I; D375G	-		-	-		-	-	
GASAR0049	1	-	-		T237I; D375G	-		-	-		-	-	
GASAR0052	1	-	-		T237I; D375G	-		-	-		-	-	
GASAR0065	1	-	-		T237I; D375G	-		-	-		-	-	
GASAR0069	1	-	-		T237I; D375G	-		-	-		-	-	
GASAR0082	1	-	-		T237I; D375G	-		-	-		-	-	
GASAR0086	1	-	-		T237I; D375G	-		-	-		-	-	
GASAR0090	1	-	-		T237I; D375G	-		-	-		-	-	
GASAR0092	1	-	-		T237I; D375G	-		-	-		-	-	
GASAR0093	1	-	-		T237I; D375G	-		-	-		-	-	
GASAR0098	1	-	-		T237I; D375G	-		-	-		-	-	
GASAR0099	1	-	-		T237I; D375G	-		-	-		-	-	
GASAR0110	1	-	-		T237I; D375G	-		-	-		-	-	
GASAR0112	1	-	-		T237I; D375G	-		-	-		-	-	
GASAR0005	2	-	-		-	-		-	-		-	-	

GASAR0014	2	-	-	-	-	-	-	-	-
GASAR0059	2	-	-	-	-	-	-	-	-
GASAR0068	2	-	-	-	-	-	-	-	-
GASAR0006	3	-	-	-	-	I37V; **192-STOP**	-	-	-
GASAR0008	3	-	-	-	-	I37V; **192-STOP**	-	-	-
GASAR0015	3	-	-	-	-	I37V; **192-STOP**	-	-	-
GASAR0034	3	-	-	-	-	I37V; **192-STOP**	-	-	-
GASAR0051	3	-	-	-	-	I37V; **192-STOP**	-	-	-
GASAR0062	3	-	-	-	-	I37V; **192-STOP**	-	-	-
GASAR0096	3	-	-	-	-	I37V; **192-STOP**	-	-	-
GASAR0026	4	Q175R	-	N222T	-	-	-	A165G	-
GASAR0040	4	Q175R	-	N222T	-	-	-	A165G	-
GASAR0073	4	Q175R	-	N222T	-	-	-	A165G	-
GASAR0102	4	Q175R	-	N222T	-	-	-	A165G	-
GASAR0028	5	-	-	-	-	A298T	-	A165G	-
GASAR0044	6	-	-	L205F	-	A279T; A298T	-	A165G	-
GASAR0070	6	-	-	L205F	-	A279T; A298T	-	A165G	-
GASAR0072	6	-	-	L205F	-	A279T; A298T	-	A165G	-
GASAR0087	6	-	-	L205F	-	A279T; A298T	-	A165G	-
GASAR0101	6	-	-	L205F	-	A279T; A298T	-	A165G	-
GASAR0106	6	-	-	L205F	-	A279T; A298T	-	A165G	-
GASAR0013	12	-	-	-	-	I37V	-	A121G; T200C	-
GASAR0030	12	-	-	-	-	I37V	-	A121G; T200C	-
GASAR0032	12	-	-	-	-	I37V	-	A121G; T200C	-
GASAR0053	12	-	-	-	-	I37V	-	A121G; T200C	-
GASAR0081	12	-	-	-	-	I37V	-	A121G; T200C	-
GASAR0103	12	-	-	-	-	I37V	-	A121G; T200C	-
GASAR0104	12	-	-	-	-	I37V	-	A121G; T200C	-
GASAR0076	22	-	-	A251D; V328M	-	I16T; A298T	-	A121G; T200C	-

GASAR0009	28	-	-	-	-	I37V	-	T181C	-
GASAR0010	28	-	-	-	-	I37V	-	T181C	-
GASAR0020	28	-	-	-	-	I37V	-	T181C	-
GASAR0036	28	-	-	-	-	I37V	-	T181C	-
GASAR0045	28	-	-	-	-	I37V	-	T181C	-
GASAR0046	28	-	-	-	-	I37V	-	T181C	-
GASAR0054	28	-	-	-	-	I37V	-	T181C	-
GASAR0055	28	-	-	-	-	I37V	-	T181C	-
GASAR0067	28	-	-	-	-	I37V	-	T181C	-
GASAR0077	28	-	-	-	-	I37V	-	T181C	-
GASAR0095	28	-	-	-	-	I37V	-	T181C	-
GASAR0111	28	-	-	-	-	I37V	-	T181C	-
GASAR0113	28	-	-	-	-	I37V	-	T181C	-
GASAR0035	43	-	-	-	-	I37V	-	-	-
GASAR0012	44	-	-	-	-	I37V	-	A165G	-
GASAR0018	44	-	-	-	-	I37V	-	A165G	-
GASAR0083	44	-	-	-	-	I37V	-	A165G	-
GASAR0061	58	E120G	-	T237I	-	L30Q; T380A	-	A165G	-
GASAR0023	75	-	-	T237I	-	-	-	G114A; A165G	-
GASAR0025	75	-	-	T237I	-	-	-	G114A; A165G	-
GASAR0063	75	-	-	T237I	-	-	-	G114A; A165G	-
GASAR0085	75	-	-	T237I	-	-	-	G114A; A165G	-
GASAR0089	75	-	-	T237I	-	-	-	G114A; A165G	-
GASAR0109	75	-	-	T237I	-	-	-	G114A; A165G	-
GASAR0038	81	Q175R	-	-	-	-	-	A165G	-
GASAR0007	87	-	-	-	-	-	-	A165G	-
GASAR0002	89	-	-	-	-	I37V	-	C156T	-
GASAR0024	89	-	-	-	-	I37V	-	C156T	-
GASAR0031	89	-	-	-	-	I37V	-	C156T	-
GASAR0037	89	-	-	-	-	I37V	-	C156T	-

GASAR0050	89	-	-	-	-	I37V	-	C156T	-
GASAR0057	89	-	-	-	-	I37V	-	C156T	-
GASAR0058	89	-	-	-	-	I37V	-	C156T	-
GASAR0064	89	-	-	-	-	I37V	-	C156T	-
GASAR0091	89	-	-	-	-	I37V	-	C156T	-
GASAR0100	89	-	-	-	-	I37V	-	C156T	-
GASAR0017	104	-	-	S298L	-	I37V; A298T; T380A	-	-	-
GASAR0105	134	E120G	-	Y231C; T237I	-	L30Q; T380A	-	A165G	-

Supplementary Table 5. Short read assembly statistics

Sample	SRA accession	Total Length	No. of Contigs	Avg Contig Length	Largest Contig	N50
GASAR0001	ERS379344	1800855	20	90042.75	698066	221515
GASAR0002	ERS361729	1753245	22	79692.95	487774	178167
GASAR0003	ERS361804	1799801	15	119986.73	817058	306993
GASAR0005	ERS379345	1896083	20	94804.15	313472	170344
GASAR0006	ERS361792	1861292	25	74451.68	516854	176303
GASAR0007	ERS361812	1866854	38	49127.74	412707	130608
GASAR0008	ERS361800	1881861	31	60705.19	251356	195302
GASAR0009	ERS361731	1894483	16	118405.19	537065	432303
GASAR0010	ERS361802	1862053	15	124136.87	503499	236456
GASAR0011	ERS361803	1800734	20	90036.7	698542	203788
GASAR0012	ERS361817	1842928	12	153577.33	383104	252730
GASAR0013	ERS361818	1837941	37	49674.08	217667	163041
GASAR0014	ERS379346	1893354	14	135239.57	317472	223989
GASAR0015	ERS379331	1819827	19	95780.37	516491	228022
GASAR0016	ERS361813	1798302	18	99905.67	695636	231567
GASAR0017	ERS361734	1723981	19	90735.84	434004	171713
GASAR0018	ERS361793	1842977	12	153581.42	325592	238078
GASAR0019	ERS379330	1802101	19	94847.42	698056	208893
GASAR0020	ERS361742	1845845	13	141988.08	504268	432304
GASAR0021	ERS379347	1800943	15	120062.87	818158	307005
GASAR0022	ERS361740	1800525	16	112532.81	816964	320034
GASAR0023	ERS361739	1860954	24	77539.75	380462	180816
GASAR0024	ERS379332	1737531	17	102207.71	495058	161261
GASAR0025	ERS361737	1858612	12	154884.33	592433	380001
GASAR0026	ERS361736	1881488	34	55337.88	320137	115096
GASAR0028	ERS361816	1850722	22	84123.73	285671	254450
GASAR0029	ERS379333	1801515	20	90075.75	698547	208887
GASAR0030	ERS379348	1839772	25	73590.88	220154	108186
GASAR0031	ERS361791	1778485	16	111155.31	488978	178840
GASAR0032	ERS379334	1758106	22	79913.91	365804	131109
GASAR0033	ERS361750	1801206	16	112575.38	751806	209783

GASAR0034	ERS379335	1860469	19	97919.42	516487	197943
GASAR0035	ERS379349	1821394	23	79191.04	278433	149332
GASAR0036	ERS361822	1854553	14	132468.07	545576	232715
GASAR0037	ERS361747	1686938	12	140578.17	629811	271014
GASAR0038	ERS361823	1757276	20	87863.8	320345	193172
GASAR0040	ERS379350	1825300	20	91265	341272	143871
GASAR0042	ERS379336	1800903	19	94784.37	698451	203792
GASAR0044	ERS361752	1882911	34	55379.74	247397	130884
GASAR0045	ERS379351	1889451	12	157454.25	532672	213754
GASAR0046	ERS361825	1846681	12	153890.08	503359	433233
GASAR0047	ERS379352	1801297	15	120086.47	698582	320074
GASAR0048	ERS361755	1807287	18	100404.83	698262	232380
GASAR0049	ERS379353	1800265	16	112516.56	817198	208720
GASAR0050	ERS361757	1820979	24	75874.12	420955	178215
GASAR0051	ERS379343	1861426	26	71593.31	516856	176298
GASAR0052	ERS379354	1801469	13	138574.54	911203	911203
GASAR0053	ERS361796	1796412	22	81655.09	242200	182665
GASAR0054	ERS361820	1888835	13	145295	532593	263156
GASAR0055	ERS361815	1851397	9	205710.78	542564	322037
GASAR0057	ERS361797	1710732	12	142561	656587	219455
GASAR0058	ERS361807	1686787	11	153344.27	630080	271072
GASAR0059	ERS361760	1907188	20	95359.4	315831	244498
GASAR0061	ERS361761	1948707	13	149900.54	481456	323032
GASAR0062	ERS361806	1859807	24	77491.96	516491	227944
GASAR0063	ERS361762	1859201	12	154933.42	491469	264926
GASAR0064	ERS361763	1728872	12	144072.67	629996	271107
GASAR0065	ERS361805	1801075	19	94793.42	698160	274131
GASAR0067	ERS361824	1812251	12	151020.92	503427	459651
GASAR0068	ERS379337	1948905	20	97445.25	416996	287621
GASAR0069	ERS361808	1801051	17	105944.18	408612	203631
GASAR0070	ERS361795	1885238	37	50952.38	261566	133329
GASAR0072	ERS379355	1853081	23	80568.74	247447	144578
GASAR0073	ERS361766	1872073	45	41601.62	399815	148449
GASAR0076	ERS361768	1763744	24	73489.33	259444	133358

GASAR0077	ERS361787	1855834	18	103101.89	499997	213538
GASAR0081	ERS361769	1796585	23	78112.39	223292	159099
GASAR0082	ERS361770	1798924	17	105819.06	697641	390896
GASAR0083	ERS361771	1842858	13	141758.31	325493	238078
GASAR0085	ERS361772	1925702	11	175063.82	470367	264942
GASAR0086	ERS379338	1801630	18	100090.56	698569	208896
GASAR0087	ERS361773	1819550	29	62743.1	245553	144142
GASAR0089	ERS361789	1859259	15	123950.6	531229	264064
GASAR0090	ERS361774	1799650	19	94718.42	751714	327980
GASAR0091	ERS361775	1713695	13	131822.69	656609	237223
GASAR0092	ERS379339	1800568	20	90028.4	697809	208886
GASAR0093	ERS361777	1800751	19	94776.37	424753	273311
GASAR0095	ERS379341	1984275	16	124017.19	578506	214300
GASAR0096	ERS361811	1858779	20	92938.95	516445	196888
GASAR0098	ERS361780	1799022	17	105824.82	697916	327841
GASAR0099	ERS361790	1800369	13	138489.92	751818	431601
GASAR0100	ERS361781	1753127	13	134855.92	465302	178220
GASAR0101	ERS379342	1886730	37	50992.7	244616	133078
GASAR0102	ERS361799	1850545	19	97397.11	352038	255347
GASAR0103	ERS379356	1838206	25	73528.24	287423	181675
GASAR0104	ERS361784	1797164	25	71886.56	384021	178821
GASAR0105	ERS361785	1745688	6	290948	825458	434381
GASAR0106	ERS361786	1885132	31	60810.71	245488	143114
GASAR0109	ERS361735	1819205	11	165382.27	550776	380384
GASAR0110	ERS361810	1799194	14	128513.86	751437	343125
GASAR0111	ERS361788	1888547	11	171686.09	503428	309434
GASAR0112	ERS361821	1838080	19	96741.05	468733	197708
GASAR0113	ERS361809	1854149	11	168559	545574	432184
